# Analysing premature cardiovascular disease mortality in the United States by obesity status and educational attainment

**DOI:** 10.1186/s12916-024-03752-x

**Published:** 2024-11-14

**Authors:** Han Li, Tim Adair

**Affiliations:** 1 Centre for Actuarial Studies, Faculty of Business and Economics, The University of Melbourne, Level 3, Building 105, 111 Barry Street, Melbourne, VIC 3010 Australia; 2 Nossal Institute for Global Health, Melbourne School of Population and Global Health, The University of Melbourne, Level 2, 32 Lincoln Square North, Melbourne, VIC 3010 Australia

**Keywords:** Cardiovascular diseases, Mortality, Multiple causes of death, United States, Obesity, Education

## Abstract

**Background:**

In the United States (US), premature cardiovascular disease (CVD) mortality rates (35–74 years) have exhibited increases in recent years, particularly in younger adults, and large differentials by educational attainment. This trend has occurred concurrently with high and increasing obesity prevalence, which also show significant differences by education. This study aims to jointly model premature CVD mortality trends in the US according to obesity status and educational attainment.

**Methods:**

We used multiple cause of death data from the National Center for Health Statistics, obesity prevalence data from the National Health and Nutrition Examination Survey (NHANES), and educational attainment data from the American Community Survey and NHANES. We applied Bayes’ theorem to these data to calculate the conditional probability of premature CVD mortality given obesity status and educational attainment for 2003–2019. We then projected this conditional probability for 2020–2029 using the Lee-Carter model.

**Results:**

The probability of premature CVD mortality was greatest for obesity and low education (not graduated high school) and was substantially higher (females 6.7 times higher, males 5.9) compared with non-obesity and high education (Bachelor’s degree or higher) in 2019. There was a widening of the gap in premature CVD mortality from 2003 to 2019 between the obese and non-obese populations, which occurred at each education level and was projected to continue in 2020–2029, especially for males. The conditional probability of premature CVD death for obesity and middle education (finished high school but no Bachelor’s degree) increased substantially and was projected to surpass the level for non-obesity and low education in coming years for males and in younger age groups. At high education, the conditional probability of premature CVD death for the obese population was projected to increase to 2029, while for non-obesity it was projected to remain steady for females and fall for males; this projected widening is greatest at older age groups.

**Conclusions:**

The findings demonstrate the public health challenge to reduce premature US CVD mortality posed by continued high obesity prevalence, especially for younger ages, lower education groups and males. The relative importance of obesity in influencing premature CVD mortality trends has risen partly due to the decline in CVD mortality attributable to other risk factors.

**Supplementary Information:**

The online version contains supplementary material available at 10.1186/s12916-024-03752-x.

## Background

A striking feature of population health in several high-income countries in recent years has been the slowdown in the long-term decline in premature cardiovascular disease (CVD) mortality [1, 2]. Such trends have been particularly poor in the United States (US), where premature CVD mortality rates (35–74 years) increased in the second half of the 2010s, particularly in younger adults [3]. Adverse trends in CVD mortality pose significant challenges for public health policy, given that it is the leading cause of death globally and was a significant contributor to improvements in life expectancy in earlier decades [4, 5].

The role of both obesity and education status in premature CVD mortality trends in the US is of much interest given the existing evidence of their relationship. There has been a sharp increase in obesity prevalence in the US in recent decades, with the National Health and Nutrition Examination Survey (NHANES) showing an increase among adults from 23% in 1988–1994 to 42% in 2017–2020 and it is now the highest-ranked of all high-income countries [6–8]. The heightened risk of mortality from obesity, including the long-term effects of childhood obesity, has been identified by several studies, particularly for CVD [9–13]. Analysis of mortality data in the US has concluded that obesity has contributed to the reversal in the decline of CVD mortality rates [3].

Recent US mortality levels have also exhibited disparities between different education groups. Lower educational attainment, especially not having completed high school, has been found to be independently associated with increased risk of both all-cause and CVD mortality in the US [14]. Such educational differences in mortality have also been revealed elsewhere, including as a proxy for socioeconomic status [15–19]. Education status is important in the context of obesity-related mortality; in the US, obesity prevalence is higher for people with lower education, including within specific ethnic groups [20]. Such differences in obesity prevalence by education have also been found in OECD countries, with larger gaps found for women compared with men [21].

In summary, in recent years the US has been experiencing adverse premature CVD mortality trends, high and increasing obesity prevalence, and significant education differentials in both. However, to date there has not been a published analysis of premature CVD mortality trends jointly according to obesity and education. Hence, in this study we fill this knowledge gap by modelling trends in the conditional probability of premature CVD mortality in the US given obesity status and educational attainment. We also project the conditional probability of premature CVD mortality according to obesity status and education to 2029; this can not only help understanding of their future trends given historical data but also provide baseline scenarios in the context of the changing landscape of pharmacological interventions to address obesity due to the increased usage of semaglutide drugs that can lead to weight loss [22].

## Methods

The study used several data sources to calculate the conditional probability of CVD mortality given obesity and education level. The mortality data used were from the National Center for Health Statistics (NCHS) Multiple Cause of Death Data file for deaths occurring in 2003–2019 [23]. We chose 2019 as the final year of data given that the significant mortality impact of the COVID-19 pandemic in the US was a outlier compared with longer-term trends [24]. This dataset comprises information of all registered deaths in the US, including all diseases and conditions reported on the death certificate. We used the dataset’s information on the decedent of age at death, sex, year of death and education level. We defined premature mortality as death from 35 to 74 years. We measured CVD mortality as a death with any CVD reported on the death certificate, excluding cardiac arrest (International Classification of Diseases, 10th Revision (ICD-10) codes I00–I45, I47–I99) [25]. We defined obesity-related mortality as CVD mortality with at least one of the following conditions reported in either part 1 (diseases and conditions in the chain of events directly leading to death) or part 2 (other significant conditions contributing to death) of the death certificate: diabetes (ICD-10 codes E10–E14), chronic kidney disease (N18), obesity (E65–E66), lipidemias (E78) and hypertension (I10-I15) [3, 25, 26]. Obesity is associated with increased risk of mortality from these causes [10, 12, 27, 28].

Annual population data were obtained from the Surveillance, Epidemiology and End Results (SEER). The SEER population data are produced by the US Census Bureau’s Population Estimates Program. Population data were collated by age for the period 2003–2019 [29]. Data of the educational attainment of the population 18 years and over were obtained from the American Community Survey (ACS), a demographic survey program conducted by the US Census Bureau of 3.5 million households each year. Educational attainment data was available for the period 2003–2019 [30]. Survey weights were used to produce these education estimates for the population. Additional file 1: Tables S1–S3 show the education classification in the NCHS data and ACS data.

The NHANES is a survey with a complex multistage and clustered sample design. It combines interviews and physical examinations, from which information on standardised measurements of weight and height are provided [31]. Body mass index (BMI) is calculated as weight in kilograms divided by height in squared metres. Obesity is defined as a BMI greater than or equal to 30 kg/m^2^. To produce nationally representative statistics on obesity, we applied survey weights to the raw data to account for oversampling, survey non-response and post-stratification adjustment. The survey includes data on individual educational attainment level (categories shown in Additional file 1: Table S4). We compiled data from the NHANES waves 2003–2004, 2005–2006, 2007–2008, 2009–2010, 2011–2012, 2013–2014, 2015–2016, 2017–2018 and 2017–March 2020 pre-pandemic data. The sample size varies with each wave—for example it was 16,211 respondents in 2017–2018.

We calculated the conditional probability of CVD mortality given obesity status and level of educational attainment. This conditional probability was calculated based on Bayes’ theorem, for a certain age and sex, as:

$$\text{Pr}(CVD|O,E)=\frac{\text{Pr}\left(O,E|CVD\right)\text{Pr}(CVD)}{\text{Pr}(O|E)\text{Pr}(E)}$$

where *CVD* denotes CVD death, *O* denotes obesity status has a value of 0 (no obesity) or 1 (obesity), and *E* denotes educational attainment level which has a value of 1 (low education level), 2 (medium education level) or 3 (high education level).

Pr(*O*,*E*|*CVD*) was computed from NCHS data as the number of CVD deaths for a given obesity status (i.e. using obesity-related CVD mortality, as defined earlier, as a proxy for CVD mortality among the obese population) and education level, divided by the total number of CVD deaths. Pr(*CVD*) was calculated as the number of CVD deaths, also from NCHS data, divided by the total population exposure (from SEER data). The denominator Pr(*O*|*E*) Pr(*E*) is the product of the proportion of the population at a given education level that are obese (from NHANES data) and the proportion of individuals in the population within a certain education level (from ACS data); it is otherwise the joint probability function Pr(*O*,*E*). This is also described in Additional file 1: Text S1.

These data sources were used to calculate each term for each age group, sex and year. Each term was then used to calculate the conditional probability of CVD mortality given obesity status and level of educational attainment—Pr(*CVD*|*O*,*E*)—for each age group, sex and year. We next fitted these mortality time series using the Lee-Carter model (1992). The model was specified as follows:

$$\text{log}\left({m}_{x,t}\right)={a}_{x}+{b}_{x}{\kappa }_{t}+{\varepsilon }_{x,t},$$

where ${m}_{x,t}$ is the mortality rate at age *x* and time *t*, calculated by the ratio of death counts to population exposure. ${a}_{x}$ and ${b}_{x}$ are age-related effects, and ${\kappa }_{t}$ represents time-related effect. ${\varepsilon }_{x,t}$ is the residual term with mean equal to 0. The Lee-Carter model incorporates both age and time factors in a single model. To estimate the model, the following constraints are necessary for identification purposes:

$$\sum_{x}{b}_{x}=1, \sum_{t}{\kappa }_{t}=0.$$

These constraints imply that ${a}_{x}$ is estimated by the average of $\text{log}\left({m}_{x,t}\right)$ over time:

$${a}_{x}=\frac{1}{T}\sum_{t=1}^{T}\text{log}\left({m}_{x,t}\right).$$

For each combination of obesity status and education level, we fitted a separate Lee-Carter model, also separately for both males and females [32].

Based on the fitted Lee-Carter models, we projected future CVD mortality for different age groups and each sex, conditional on each combination of obesity status and educational attainment level. To project future mortality rates, a time series model was fitted to ${\kappa }_{t}$; Lee and Carter suggested that a random walk with drift process is most appropriate for mortality projections [32]. A random walk with drift process was specified as follows:

$${\kappa }_{t}={\kappa }_{t-1}+d+{e}_{t},$$

where $d$ is the drift term, and ${e}_{t}$ is the error term with mean equal to 0 and standard deviation equal to *σ*. The random walk with drift model is also known as the ARIMA (0,1,0) model. The mortality projection was implemented via the R package ‘demography’ [33].

## Results

**Fig. 1:**
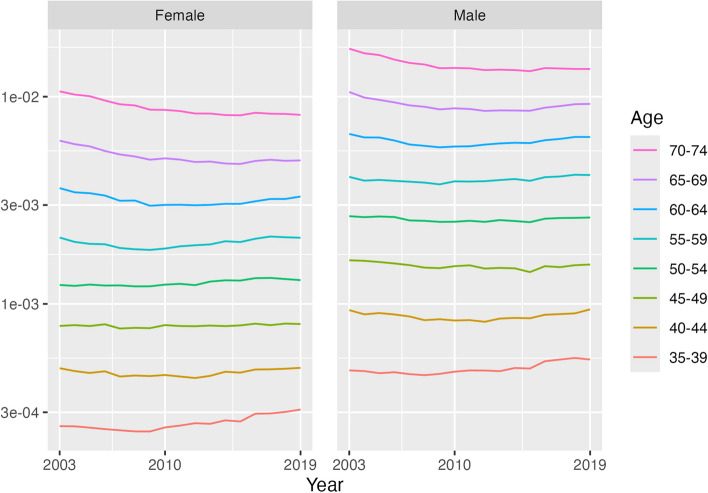
CVD mortality rate trends (log scale), by sex, 35–74 years, US, 2003–2019. Note: This chart shows Pr(*CVD*) in the conditional probability equation. The vertical axis is on a log scale. Source: NCHS data

Figure 1 and Table 1 present the trend in the CVD mortality rate by age and sex from 2003 to 2019 (i.e. where a CVD excluding cardiac arrest has been reported on the death certificate). An overall pattern with the CVD mortality rate trends was that they were worse in the most recent years and at younger ages. From 2003 to 2011, the annual rate of change in CVD mortality reached − 2.7% per annum (95% credible interval − 2.9 to − 2.5%) in the 70–74 years age group and showed a decline in almost every age group for each sex. However, in 2011–2019 small declines were only found for the 65–69 (females) and 70–74 years (both sexes) age groups, while there were increases in most other age groups. These rises included more than 1% per annum in several age groups and reached 2.2% (1.6 to 2.9%) for females aged 35–39 and 1.5% (1.0 to 2.0%) in males aged 35–39 years. In most age groups up to 55–59 years, the CVD mortality rate was higher in 2019 compared with 2003, most notably in the 35–39 years age group (female: 1.2% per annum (0.8 to 1.5%), equivalent to 20% higher in 2019 compared with 2003; male: 0.8% per annum (0.5 to 1.0%), 13% higher in 2019 compared with 2003).

**Table 1:** Annualised rate of change (%) in CVD mortality rate, by sex and age group, 35–74 years, US, 2003–2011, 2011–2019, 2003–2019

Year/sex	Age group							
	**35**–**39**	**40**–**44**	**45**–**49**	**50**–**54**	**55**–**59**	**60**–**64**	**65**–**69**	**70**–**74**
Female								
2003–2011	0.1 (− 0.6 to 0.8)	− 1.2 (− 1.6 to − 0.7)	0.0 (− 0.4 to 0.4)	0.2 (− 0.1 to 0.5)	− 1.2 (− 1.5 to − 1.0)	− 2.3 (− 2.5 to − 2.1)	− 2.6 (− 2.8 to − 2.4)	− 2.7 (− 2.9 to − 2.5)
2011–2019	2.2 (1.6 to 2.9)	1.2 (0.7 to 1.7)	0.3 (− 0.1 to 0.7)	0.5 (0.2 to 0.8)	1.2 (1.0 to 1.4)	1.1 (1.0 to 1.4)	− 0.1 (− 0.3 to 0.1)	− 0.5 (− 0.7 to − 0.4)
2003–2019	1.2 (0.8 to 1.5)	0.0 (− 0.2 to 0.3)	0.1 (− 0.1 to 0.3)	0.3 (0.2 to 0.5)	0.0 (− 0.1 to 0.1)	− 0.6 (− 0.7 to − 0.5)	− 1.4 (− 1.5 to − 1.3)	− 1.6 (− 1.7 to − 1.5)
Male								
2003–2011	0.0 (− 0.5 to 0.5)	− 1.4 (− 1.7 to − 1.0)	− 0.7 (− 1.0 to − 0.4)	− 0.6 (− 0.9 to − 0.4)	− 0.6 (− 0.8 to − 0.5)	− 1.7 (− 1.9 to − 1.5)	− 2.4 (− 2.5 to − 2.2)	− 2.7 (− 2.8 to − 2.6)
2011–2019	1.5 (1.0 to 2.0)	1.5 (1.1 to 1.9)	0.1 (− 0.2 to 0.4)	0.5 (0.2 to 0.7)	0.9 (0.7 to 1.1)	1.3 (1.1 to 1.4)	0.7 (0.6 to 0.8)	− 0.1 (− 0.2 to 0.0)
2003–2019	0.8 (0.5 to 1.0)	0.1 (− 0.1 to 0.2)	− 0.3 (− 0.4 to − 0.2)	− 0.1 (− 0.2 to 0.0)	0.1 (0.0 to 0.2)	− 0.2 (− 0.3 to − 0.1)	− 0.8 (− 0.9 to − 0.8)	− 1.4 (− 1.5 to − 1.3)

Source: NCHS data. 95% confidence intervals shown in parentheses

Between 2003–2010 and 2011–2019, there was an increase in the percentage of each age group’s population that were obese (Additional file 1: Table S5). The increase ranged from 2.3 percentage points for males aged 65–69 years (2003–2010 36.6%; 2011–2019 38.9%) to 7.3 percentage points for females aged 70–74 years (2003–2010 37.0%; 2011–2019 44.3%). The level of obesity was similar across age groups in 2011–2019, ranging by 3.7 percentage points for females (40–44 years 41.3%; 65–69 years 45.0%) and 4.0 percentage points for males (70–74 years 37.1%; 45–49 years 41.1%).

Additional file 1: Fig. S1 (Pr(*E*) in the conditional probability equation) shows that there was a clear decline in the proportion of the younger population with middle educational attainment (completed high school but no Bachelor’s degree) (35–39 years: females 2003 58.5%, 2019 45.9%; males 2003 57.0%, 2019 51.6%), which was offset by an increase in the proportion with high education (Bachelor’s degree or higher), especially for females (35–39 years: females 2003 30.8%, 2019 45.8%; males 2003 28.9%, 2019 37.9%). At older ages, more males than females (70–74 years, 2019: females 30.3%; males 39.5%) had high educational attainment, while middle educational attainment remained steady as the most common level (70–74 years, 2019: females 58.6%; males 51.4%). There was an increase in the proportion of the population with high educational attainment (70–74 years: females 2003 14.2%, 2019 30.3%; males 2003 23.8%, 2019 39.5%) offset by declines in low education (did not complete high school) (70–74 years: females 2003 28.2%, 2019 11.1%; males 2003 14.2%, 2019 10.5%). Only approximately one-tenth of those in younger age groups had a low level of educational attainment (35–39 years, 2019: females 8.3%; males 10.5%).

A higher proportion of people who attained low or middle education were obese compared with those of higher education (Additional file 1: Fig. S2; Pr(*O*|*E*) in the conditional probability equation). At most age groups, the level of obesity in the low or middle education groups was around one-third higher than for high education (e.g. 50–54 years, 2011–2019, females: low and middle 48.1%; high 32.4%). The proportion of the population who were obese peaked at ages 55–59 or 60–64 years. However, for females of high education during 2011–2019, obesity was far lower at ages 35–39 (30.5%) and 40–44 years (30.9%) than 70–74 years (35.2%), unlike for females of low-middle education (35–39 years 48.2%; 70–74 47.2%) and for males of both education categories (high: 35–39 years 33.3%, 70–74 32.4%; low-middle: 35–39 42.9%, 70–74 40.2%). The increase in obesity from 2003–2010 to 2011–2019 was fairly consistent, with the exception of a large rise from a low base among younger high education females (35–39 years: 2003–2010 19.3%; 2011–2019 30.5%).

**Fig. 2:**
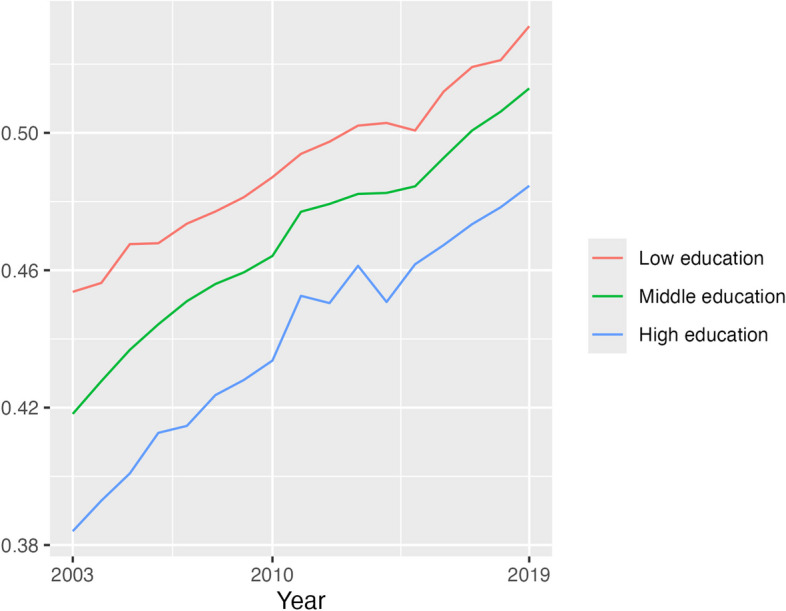
Proportion of CVD deaths at each education level that were obesity-related, females, 35–74 years, US, 2003, 2010 and 2019. Source: NCHS data

**Fig. 3:**
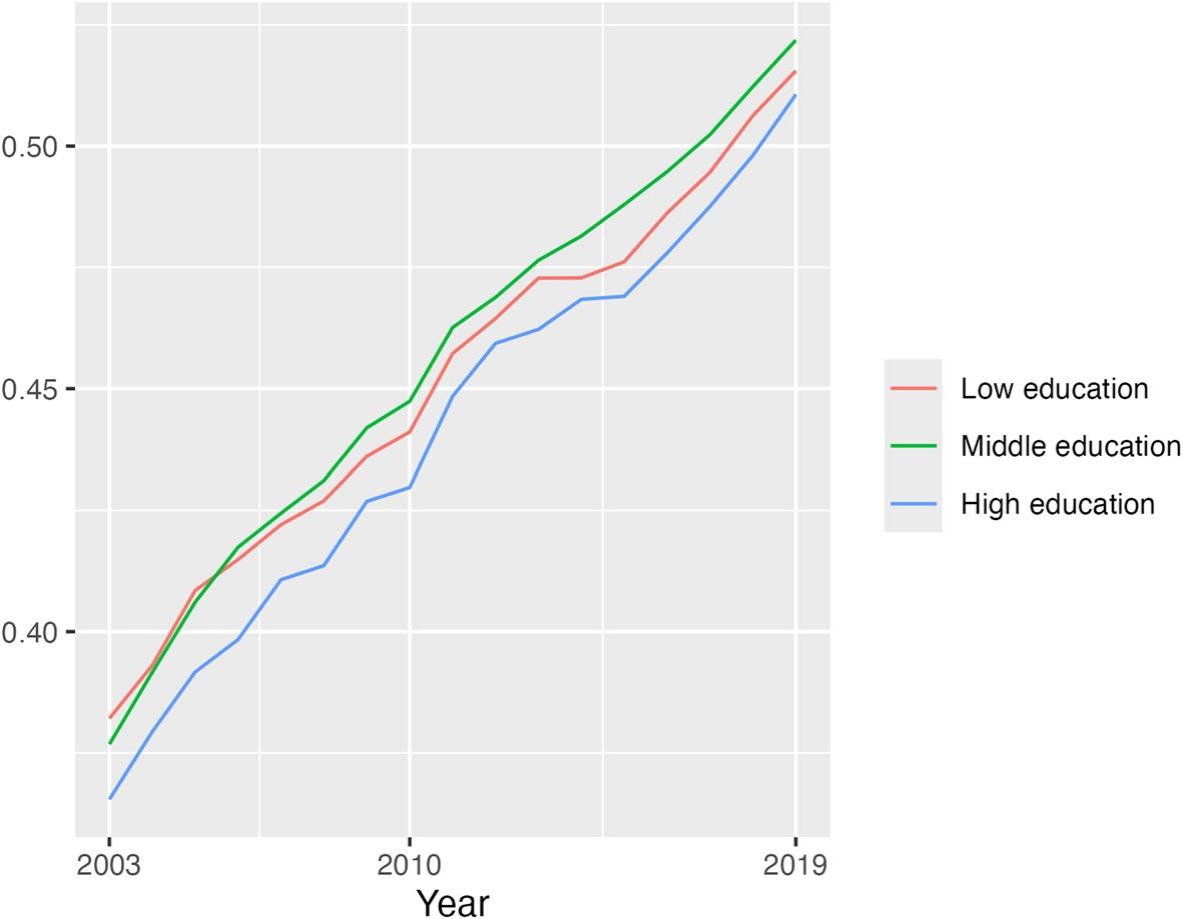
Proportion of CVD deaths at each education level that were obesity-related, males, 35–74 years, US, 2003, 2010 and 2019. Source: NCHS data

Concurrent with the rising prevalence of obesity in the US population was the increasing percentage of premature CVD deaths that were obesity-related (Figs. 2 and 3). This rise was consistent for both females and males and at all education levels. The increase in obesity-related CVD deaths was at least 10 percentage points from 2003 to 2019, reaching at least 50% of all CVD deaths by the end of the period for all groups except females of high education (48.5%).

Additional file 1: Figs. S3–S4 show the proportion of CVD deaths within each obesity and education category. These reflect trends in education in Additional file 1: Fig. S1 and in obesity-related CVD mortality in Figs. 2 and 3. At each age group, there was a decline in the proportion of CVD deaths that were not related to obesity and that were in the low education category (e.g. females 55–59 years: 2003 12.5%, 2019 8.4%), while the trend of deaths related to obesity was inconsistent across age. For both women and men of the middle education level, the proportion of CVD deaths that were obesity-related increased over time (e.g. 65–69 years: females 2003 25.8%, 2019 35.6%; males 2003 21.1%, 2019 34.3%) while the proportion that were not obesity-related remained steady (e.g. 65–69 years: females 2003 35.3%, 2019 33.2%; males 2003 34.2%, 2019 32.2%). The proportion of CVD deaths for those of high education that were obesity-related also rose, but from a low base (e.g. 65–69 years: females 2003 3.3%, 2019 7.8%; males 2003 5.5%, 2019 8.9%), while CVD deaths for this education level that were not obesity-related exhibited inconsistent trends.

**Fig. 4:**
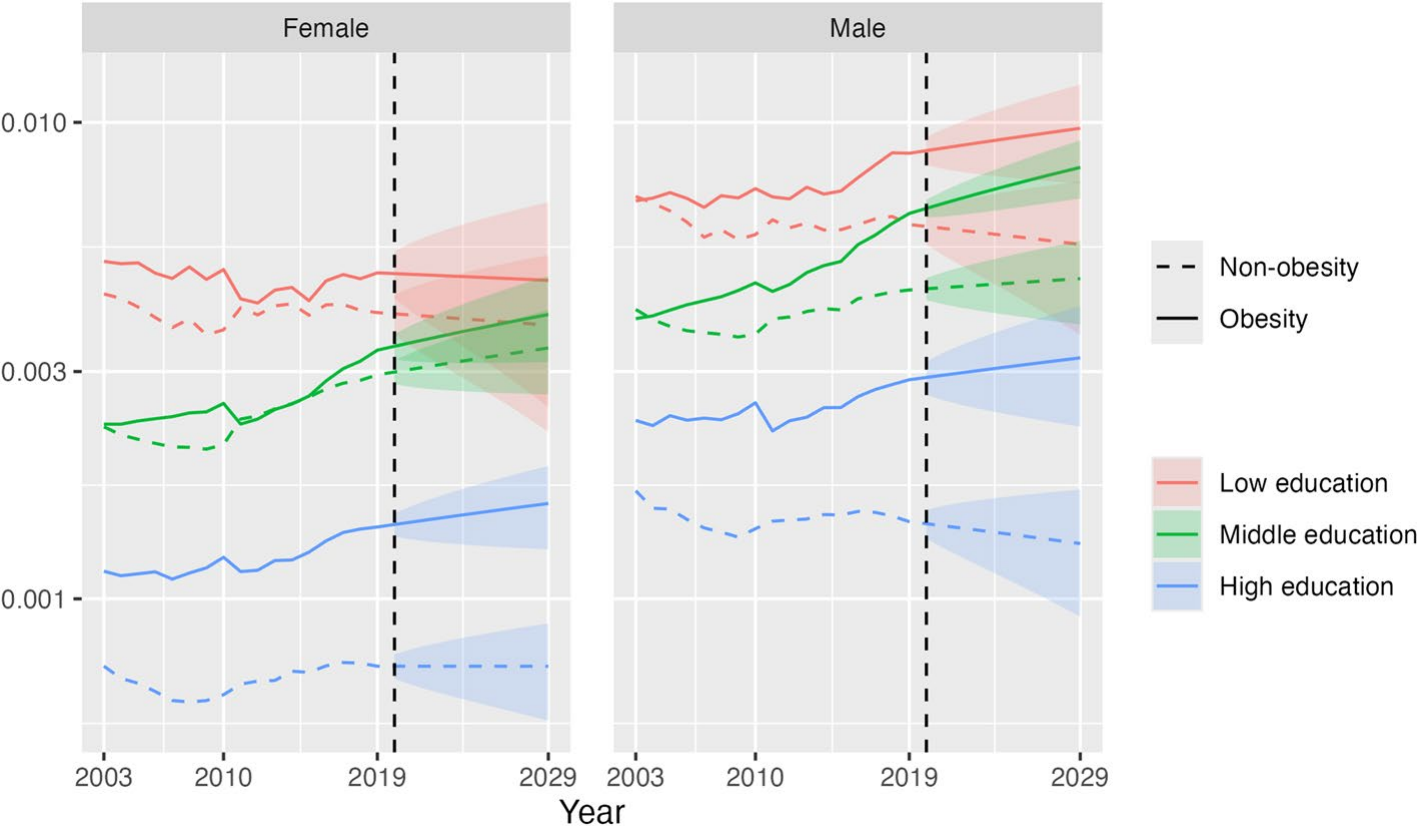
Conditional probability of CVD deaths given obesity status and educational attainment level, by education and sex, 35–74 years, US, 2003–2019 (actual) and 2020–2029 (projection). Note: This chart shows Pr(*CVD*|*O*,*E*) in the conditional probability equation. The vertical axis is on a log scale. Shaded areas are 95% prediction intervals

Figure 4 shows our main results of the conditional probability of premature CVD death for a given combination of obesity status and education category, for all ages combined, in 2003–2019 and in the projection period 2020–2029. The conditional probability of premature CVD mortality was highest for the obese population and low education and lowest for the non-obese population and high education, being 6.7 times higher for females and 5.9 times higher for males in 2019 and projected to widen further by 2029. The gap in the conditional probability between obesity and non-obesity progressively increased over 2003–2019 across each education level and is projected to continue to do so until 2029, especially for males (e.g. middle education, gap in 2019: 199 per 100,000; gap in 2029: 334 per 100,000). A notable finding is that the conditional probability of CVD death for obesity and middle education increased significantly to 2019 and is projected to continue to do so (females, 2019: 333 per 100,000; 2029: 395 (314–476)). By 2020, it had surpassed the level for low education and non-obesity for males and is projected to be clearly higher by 2029 (obesity, middle education 804 per 100,000 (692–917); non-obesity, low education 555 (358–752)), while for females it is projected to be higher in 2028, both with only minimal overlap of 95% prediction intervals (PIs) (obesity, middle 389 (314–464); non-obesity, low 378 (237–518)). At high education, the conditional probability of CVD death is relatively wide between obese and non-obese populations in 2019 and projected to continue to widen; for obesity it is projected to increase to 2029, although with some overlap of PIs (females: 2019 142 per 100,000; 2029 159 (127–190); males: 2019 288; 2029 320 (230–411)), while for non-obesity it is projected to remain steady for females (2019 72; 2029 72 (56–89)) and fall for males (2019 145; 2029 131 (92–170)).

**Fig. 5:**
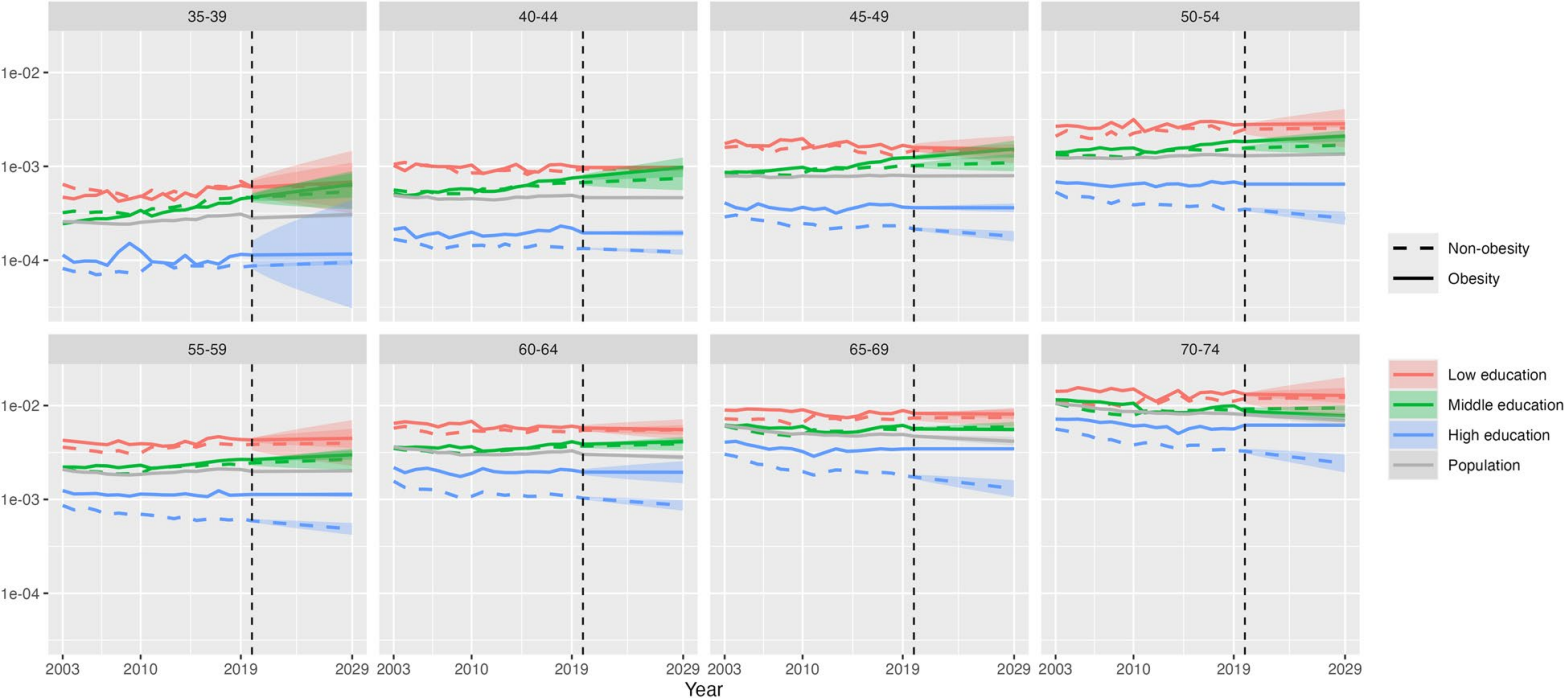
Conditional probability of CVD death given obesity status and educational attainment level, by age group and education, females, 35–74 years, 2003–2019 (actual) and 2020–2029 (projection). Note: This chart shows Pr(*CVD*|*O*,*E*) in the conditional probability equation. The vertical axis is on a log scale. Shaded areas are 95% prediction intervals

**Fig. 6:**
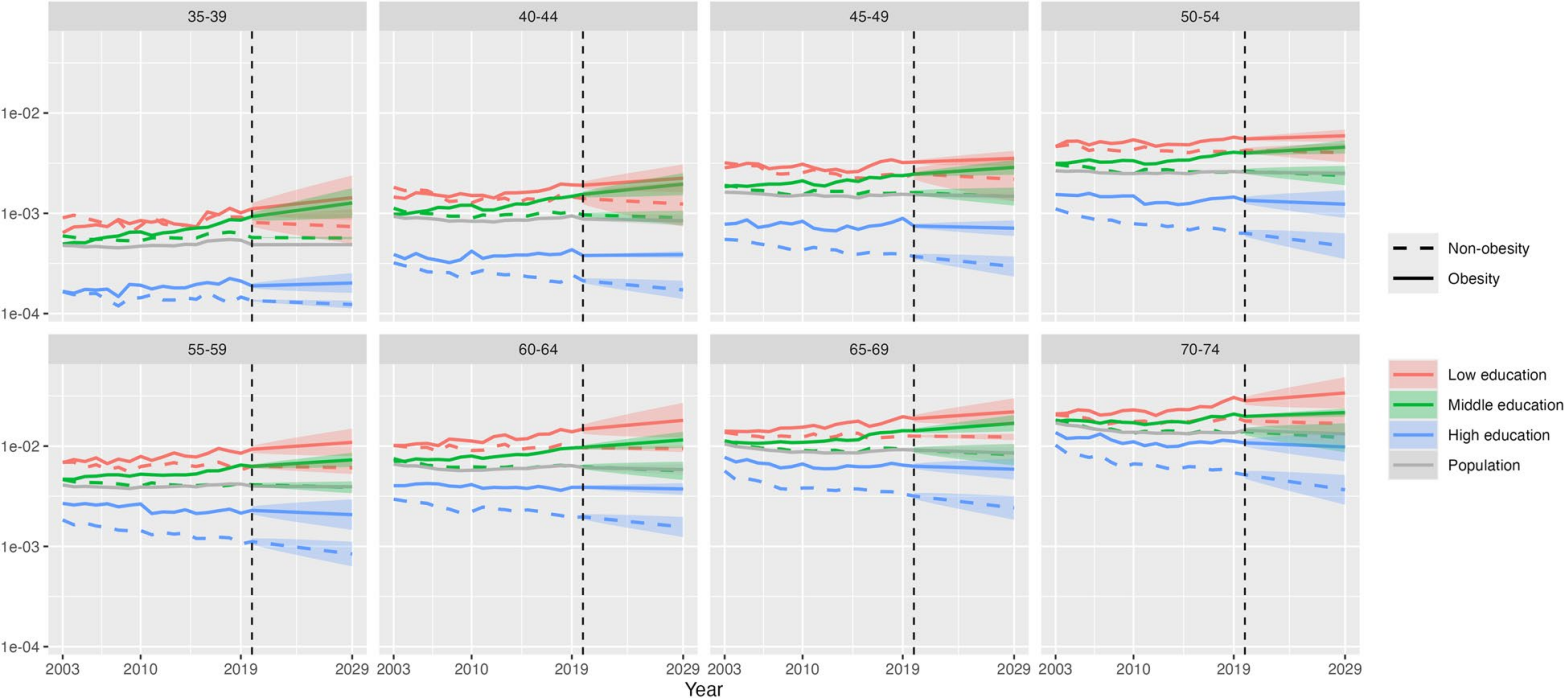
Conditional probability of US CVD deaths given obesity status and educational attainment level, by age group and education, males, ages 35–74, years 2003–2019 (actual) and 2020–2029 (projection). Note: This chart shows Pr(*CVD*|*O*,*E*) in the conditional probability equation. The vertical axis is on a log scale. Shaded areas are 95% prediction intervals

At younger age groups, the conditional probability of premature CVD mortality for obesity and middle education is projected to exceed that of non-obesity and low education by 2029, although the overlap of 95% prediction intervals means that there is some uncertainty about this finding for specific age groups. For example, at 40–44 years in 2029, the conditional probability for females with obesity and of middle education is projected to be 98 per 100,000 (77–124), while for females not obese and of low education it is projected to be 94 (89–99). For males 40–44 years, the respective figures in 2029 are 196 (152–252) and 124 (74–208) (Figs. 5 and 6). At the high education level, the projected widening of the conditional probability of CVD death between obese and non-obese populations is greatest in older age groups due to decreases in risk for the non-obese population (70–74 years, females: 2019, 24 per 100,000; 2029, 17 (14–21)), while the risk for the obese population shows only slight decreases or no change, particularly for females and in younger age groups (35–39 years, females: 2019, 21; 2029, 20 (16–25)).

## Discussion

This study has found that trends in premature CVD mortality in the US have been diverging according to obesity status and education level in recent years, which is projected to continue to 2029. The conditional probability of premature CVD mortality for the obese population has fared worse than the non-obese population across each education level, either increasing or remaining steady in recent years and projected to continue to do so. Notably, these trends for the obese population have been worse for males, at younger ages and for those who attained middle and low education levels, where the conditional probability of premature CVD is consistently projected to rise. In contrast, for the non-obese population of high education, the conditional probability of CVD mortality has been falling and is projected to continue to decline in several age groups, leading to wide divergence with the obese population of the same education level by 2029. In fact, for the male obese population of middle education, the probability of premature CVD mortality in recent years and in the projection period exceeds that for the non-obese population of low education.

The worsening premature CVD mortality among individuals with obesity compared to those without highlights the heightened risk of dying from this cause for those with a high BMI. It also reflects the trend of obesity becoming a relatively more important contributor to premature CVD mortality due to declines in the past two decades in CVD mortality attributable to other risk factors, including smoking (whose prevalence has continued to fall), dietary risks and high cholesterol [34, 35]. The relative decline in importance of these other CVD risk factors may also explain why the divergence in premature CVD mortality by obesity status is largest in the high education group, who have relatively low prevalence of these other risk factors, especially among those who are not obese [14, 36]. Additionally, improvements in diagnosis, treatment and ongoing management of CVDs have contributed to lower CVD death rates over time, for which those of high education benefit most from because they have superior access to high-quality health care compared with those of lower education [37, 38]. In contrast, those of lower education do not benefit as much from not being obese, potentially because of their relatively high prevalence levels of other risk factors for CVD and poorer access to high-quality health care.

These factors also have a role in explaining age and sex differences in the findings. There have been more favourable trends in CVD mortality rates in the older than younger age groups because of particularly strong declines in the contribution of other risk factors to CVD mortality [35]. There are wider educational differences in premature CVD mortality among the obese population at younger ages compared with older ages. A relatively higher proportion of the younger population have a high education (i.e. Bachelor’s or higher) compared with the older population, which means that in the younger population those of low or middle education are of relatively lower socioeconomic status. This translates into relatively higher premature CVD mortality risk among younger populations of low education due to them having more CVD risk factors than their higher educated peers and also reflects broader economic trends in recent decades that led to poorer income growth for those of low compared with high education [14, 39]. Compared to females, males at low and middle education levels who are obese have a relatively higher probability of premature CVD mortality than the non-obese. This is likely due to males having higher mortality from other CVD risk factors that are common among the obese population of low education, specifically daily smoking, sedentary behaviour and heavy drinking [36]. Males have also been found to have lower likelihood of seeking healthcare compared with females [40]. These factors would particularly increase CVD mortality risk for the male obese population who already are in less than ideal health. This highlights the multi-faceted challenge of narrowing sex differences in CVD mortality among the obese population of lower education.

This study’s findings have implications for population level premature CVD mortality trends. Obesity levels, including long-term obesity from childhood, are higher for the younger population than they were for the older population at the same age, so their higher risk of CVD mortality is likely to translate to increasing population-level premature CVD mortality rates [12, 13]. This trend is somewhat balanced by the low and middle education group being smaller in younger age groups (Additional file 1: Fig. S1), so a relatively smaller proportion of younger cohorts are exposed to the worsening probability of CVD mortality at these lower education levels. Notwithstanding this issue, this study’s findings highlight the urgent need for government and non-government institutions to strengthen strategies and interventions that promote healthy diet and lifestyles, especially among younger adult populations, those of lower education and males, and improve physical environments to be less obesogenic, particularly in lower socioeconomic areas [41].

The projections of the conditional probability of CVD mortality made in this study were based upon data from 2003 to 2019. Hence, they do not account for the increase in premature CVD mortality from the COVID-19 pandemic, either in the initial years of the pandemic or any longer-term effects (e.g. Long COVID). During 2020, excess mortality from overweight- and obesity-related premature CVD mortality was estimated to be 29% for males and 30% for females [26]. Our mortality projections also do not account for potential benefits from semaglutide drugs such as Ozempic and Wegovy that can lead to weight loss [22, 42]. However, accurate modelling of the mortality impact of such drugs depends on factors such as their accessibility and affordability, adherence, their longer-term impact on weight and also mortality, and how these differ across differ education groups; such modelling is beyond the scope of this project. The intention of this study instead has been to project premature CVD mortality according to obesity status based on longer-term historical data to provide baseline scenarios according to its known relationships with age and education; these are nonetheless valuable in the context of the increasing usage and potential population level benefits of semaglutide drugs. If the population of lower education have less access to the benefits of these drugs compared with higher education groups, this will exacerbate these already wide inequalities in CVD mortality.

There are other limitations with our analysis. We used an indirect measure of obesity-related CVD mortality, determined using conditions reported on the death certificate (i.e. multiple cause of death data) that have been found to have an increased risk of mortality from obesity—any mention of CVD with at least one of diabetes, chronic kidney disease, hypertensive heart disease, obesity or lipidemias—rather than CVD death specifically of a person with obesity, which our data could not measure [10, 12, 27, 28]. Four-fifths of all deaths aged 35–74 years in the US attributed to obesity and overweight have either diabetes, chronic kidney disease or CVD (which includes hypertensive heart disease) as the underlying cause; furthermore, this measurement has previously been used to measure obesity-related premature CVD mortality [3, 26, 35]. Another potential issue with this indicator is that, if there were an increased number of conditions reported on the death certificate due to changes in guidance to doctors about completing a death certificate or from improved diagnostic capabilities, there could result in artificial increases in mortality rates measured by multiple cause of death data, however trends in premature CVD mortality based on a single underlying cause and multiple cause data have been very similar in recent years [3].

Our calculation of the conditional probability of premature CVD mortality given obesity status and educational attainment was based on data collated from different sources that were combined at the level of age group, sex and year. Therefore, it is potentially subject to the ecological fallacy—bias from a calculation made about individuals from aggregated data. However, there are no available data sources with the population coverage of those we have used that enable calculation of this conditional probability of CVD mortality from individual-level data. The role of obesity prevalence and educational attainment in influencing mortality, like many factors related with mortality, occurs over the long term whereas we only measure these indicators in the year of the death. Lastly, our analysis did not include other variables that may have provided further detail for the results, including geography (e.g. urban/rural status, state) which were not included in the mortality data (and which are likely to be partly captured by educational attainment [43]), smoking status which is a risk factor for CVD mortality, and race/ethnicity for which cut-off values of BMI to measure obesity can vary [44]. It could be that some of the educational differences in the conditional probability of premature CVD mortality would be explained by urban/rural status or race/ethnicity. However, a recent article analysing US data found that lower educational attainment is associated with higher risk of CVD mortality within different race/ethnicity groups [14].

## Conclusions

The study’s findings demonstrate the public health challenge posed by high and increasing obesity prevalence in the US, especially for younger age groups, those of lower education (and hence socioeconomic status) and for males. In the context of recent increases in premature CVD mortality rates (35–74 years), particularly in younger adults, and the importance of CVD as a cause of death, these projected trends paint a concerning picture of the future trajectory of life expectancy in the US and its socioeconomic inequalities [3, 5]. This is especially apparent given the dramatic impact of the COVID-19 pandemic on mortality in the US, high and increasing levels of mortality from the opioid epidemic and other drug-related deaths, suicide and alcohol-related mortality, as well as substantial inequities in access to affordable and high-quality health care [37, 45].

## Supplementary Information

Additional file 1: Additional tables, text and figures that were not included in the main text. Table S1 NCHS 1989 version of educational attainment. Table S2 NCHS 2003 version of educational attainment. Table S3 ACS educational attainment codes. Table S4 NHANES educational attainment codes. Table S5 Percentage of the population that are obese, by sex and age group, 35–74 years, US, 2003–2010 and 2011–2019. Text S1 Description of conditional probability of CVD mortality given obesity status and level of educational attainment equation. Fig. S1 Proportion of the population in each education category, by sex and age group, 35–74 years, US, 2003–2019. Fig. S2 Proportion of the population of each educational attainment category that are obese, by sex and age group, 35–74 years, US, 2003–2010 and 2011–2019. Fig. S3 Proportion of CVD deaths within a specific obesity and education category, females, by age group, 35–74 years, US, 2003–2019. Fig. S4 Proportion of CVD deaths within a specific obesity and education category, males, by age group, 35–74 years, US, 2003–2019.

## Data Availability

The multiple cause of death data file is available from the National Center for Health Statistics (https://www.cdc.gov/nchs/nvss/mortality_public_use_data.htm), the population data are available from the Survey of Epidemiology and End Results at https://seer.cancer.gov/data-software/uspopulations.html, the American Community Survey data are available from https://www.census.gov/programs-surveys/acs/data.html, and the National Health and Nutrition Examination Survey data are available from https://wwwn.cdc.gov/nchs/nhanes.

## Abbreviations

ACS: American Community Survey
BMI: Body mass index
CVD: Cardiovascular diseases
ICD-10: International Classification of Diseases, 10th Revision
NCHS: National Center for Health Statistics
NHANES: National Health and Nutrition Examination Survey
SEER: Surveillance, Epidemiology and End Results
US: United States
