# Supplementary material for: Analysing premature cardiovascular disease mortality in the United States by obesity status and educational attainment
Source: BMC Med. 2024 Nov 14;22:533. doi: 10.1186/s12916-024-03752-x (PMC11566442; doi:10.1186/s12916-024-03752-x)
Supplement: Supplementary file 1 — Additional file 1: Additional tables, text and figures that were not included in the main text. Table S1 NCHS 1989 version of educational attainment. Table S2 NCHS 2003 version of educational attainment. Table S3 ACS educational attainment codes. Table S4 NHANES educational attainment codes. Table S5 Percentage of the population that are obese, by sex and age group, 35–74 years, US, 2003–2010 and 2011–2019. Text S1 Description of conditional probability of CVD mortality given obesity status and level of educational attainment equation. Fig. S1 Proportion of the population in each education category, by sex and age group, 35–74 years, US, 2003–2019. Fig. S2 Proportion of the population of each educational attainment category that are obese, by sex and age group, 35–74 years, US, 2003–2010 and 2011–2019. Fig. S3 Proportion of CVD deaths within a specific obesity and education category, females, by age group, 35–74 years, US, 2003–2019. Fig. S4 Proportion of CVD deaths within a specific obesity and education category, males, by age group, 35–74 years, US, 2003–2019. [file 12916_2024_3752_MOESM1_ESM.docx]

**Additional file 1: Analysing premature cardiovascular disease mortality in the United States by obesity status and educational attainment**

Table S1: NCHS 1989 version of educational attainment

| NCHS code | Description | Level |
| --- | --- | --- |
| 0 | No formal education | low |
| 01-08 | Years of elementary school | low |
| 9 | 1 year of high school | low |
| 10 | 2 years of high school | low |
| 11 | 3 years of high school | low |
| 12 | 4 years of high school | middle |
| 13 | 1 year of college | middle |
| 14 | 2 years of college | middle |
| 15 | 3 years of college | middle |
| 16 | 4 years of college | high |
| 17 | 5 years of college or above | high |

Table S2: NCHS 2003 version of educational attainment

| NCHS code | Description | Level |
| --- | --- | --- |
| 1 | 8th grade or less | low |
| 2 | 9-11^th^ grade (includes 12^th^ grade with no diploma) | low |
| 3 | High school graduate/GED or equivalent | middle |
| 4 | Some college credit but no degree | middle |
| 5 | Associate degree | middle |
| 6 | Bachelor’s degree | high |
| 7 | Master’s degree | high |
| 8 | Doctorate or professional degree | high |

Table S3: ACS educational attainment codes

| USCB description | Level |
| --- | --- |
| 1^st^ - 4^th^ grade | low |
| 5^th^ - 6^th^ grade | low |
| 7^th^ - 8^th^ grade | low |
| 9^th^ grade | low |
| 10^th^ grade | low |
| 11^th^ grade | low |
| High school graduate | middle |
| Some college no degree | middle |
| Associate degree – occupational | middle |
| Associate degree – academic | middle |
| Bachelor’s degree | high |
| Master’s degree | high |
| Professional degree | high |
| Doctorate degree | high |

Table S4: NHANES educational attainment codes

| NHANES code | Description | Level |
| --- | --- | --- |
| 1 | Less than 9^th^ grade | low |
| 2 | 9-11^th^ grade (includes 12^th^ grade with no diploma) | low |
| 3 | High school graduate/GED or equivalent | middle |
| 4 | Some college or AA degree | middle |
| 5 | College graduate or above | high |

**Table S5: Percentage of the population that are obese, by sex and age group, 35-74 years, US, 2003–2010 and 2011–2019**

| Year/Sex | Age Group | | | | | | | |
| --- | --- | --- | --- | --- | --- | --- | --- | --- |
| **Female** | **35**–**39** | **40**–**44** | **45**–**49** | **50**–**54** | **55**–**59** | **60**–**64** | **65**–**69** | **70**–**74** |
| 2003–2010 | 35.2 | 36.2 | 37.5 | 39.1 | 40.8 | 40.8 | 39.6 | 37.0 |
| 2011–2019 | 41.7 | 41.3 | 41.7 | 42.8 | 44.4 | 45.1 | 45.0 | 44.3 |
| **Male** | **35**–**39** | **40**–**44** | **45**–**49** | **50**–**54** | **55**–**59** | **60**–**64** | **65**–**69** | **70**–**74** |
| 2003–2010 | 36.2 | 35.5 | 35.3 | 35.5 | 36.9 | 37.4 | 36.6 | 34.6 |
| 2011–2019 | 39.9 | 40.7 | 41.1 | 40.7 | 40.5 | 40.0 | 38.9 | 37.1 |

Source: NHANES data.

Note: Survey weights were applied. We combined the four NHANES waves 2003–2004, 2005–2006, 2007–2008, and 2009–2010 to form estimates of obesity prevalence for the period 2003–2010. We combined the remaining five NHANES waves 2011–2012, 2013–2014, 2015–2016, 2017–2018, and 2017–March 2020 Pre-Pandemic Data to obtain estimates of obesity prevalence for the period 2011–2019.

**Text S1: Description of conditional probability of CVD mortality given obesity status and level of educational attainment equation**

**Bb**

**Cb**

**Db**

**A**

$$\Pr(CVD|O,E)=\frac{\Pr\left( O,E | CVD \right)\Pr(CVD)}{\Pr\left( O | E \right)\Pr(E)}$$

**A**: The proportion of all cardiovascular disease (CVD) deaths for a given obesity status and education level. From NCHS mortality data.

**B**: Mortality rate due to CVD, calculated as the number of CVD deaths from NCHS data divided by the total population exposure from SEER data.

**C**: The proportion of the population within each education attainment category that are obese. From NHANES data.

**D**: The proportion of individuals in the total population within a specific education category. From ACS data.

Each term was calculated for each age group, sex and year. From this, the conditional probability of CVD mortality given obesity status and level of educational attainment was calculated.

**Figure S1: Proportion of the population in each education category, by sex and age group, 35-74 years, US, 2003–2019**


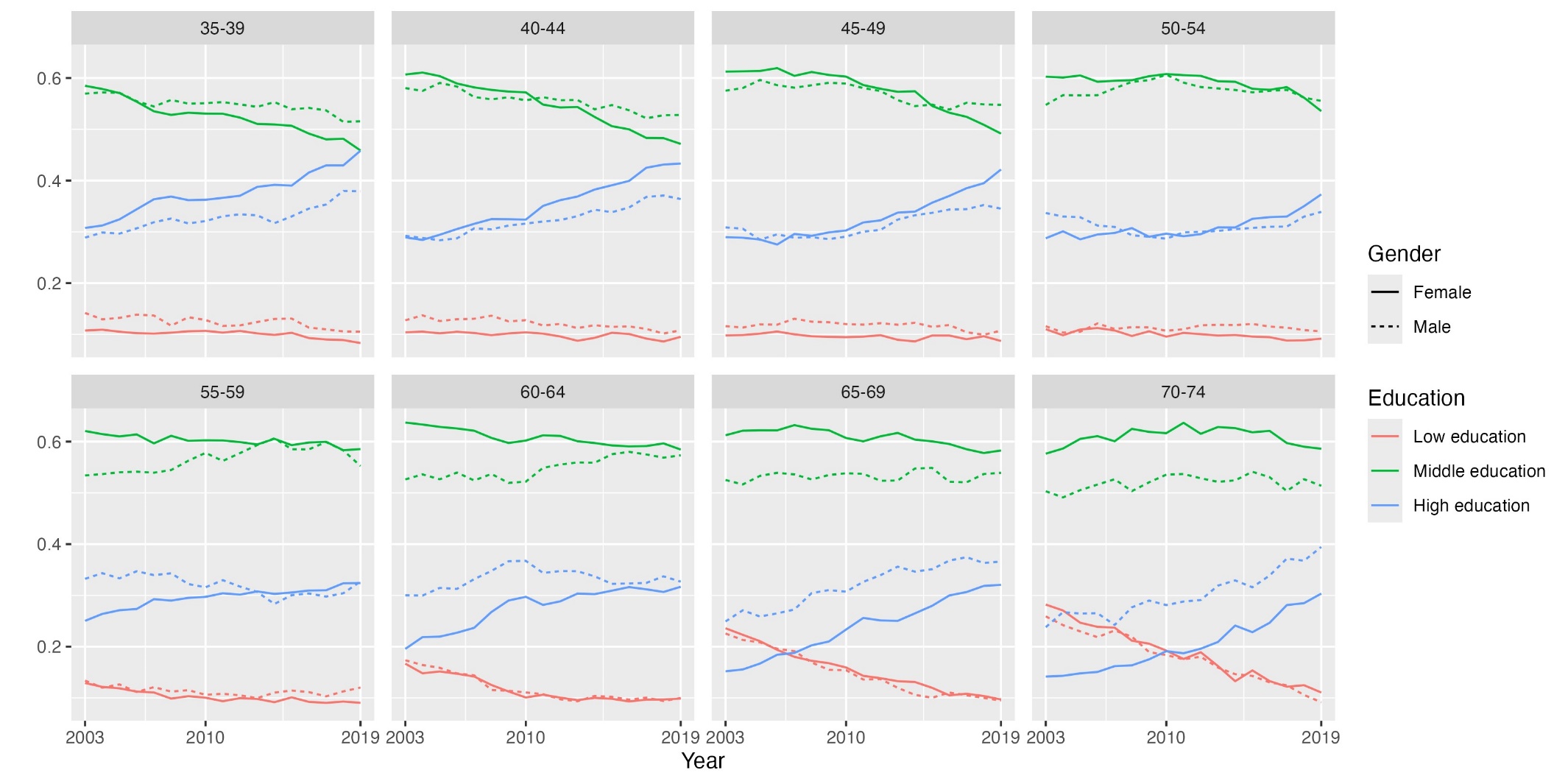


Source: ACS data. Note: This chart shows *Pr(E)* in the conditional probability equation. Survey weights were applied.

**Figure S2: Proportion of the population of each educational attainment category that are obese, by sex and age group, 35–74 years, US, 2003–10 and 2011–19**


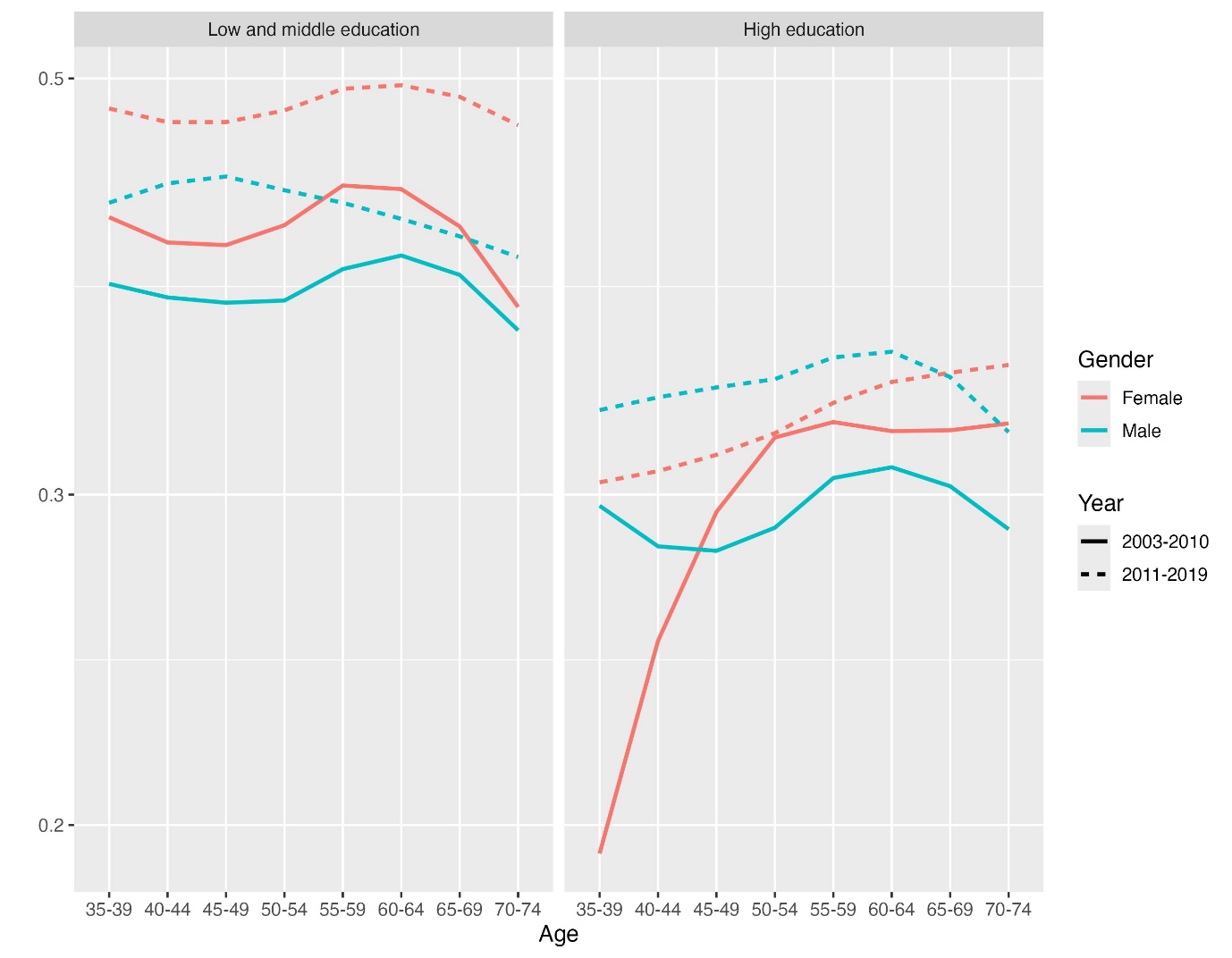


Source: NHANES data. Note: This chart shows *Pr(O|E)* in the conditional probability equation. Survey weights were applied.

**Figure S3: Proportion of all CVD deaths that were within a specific obesity and education category, females, by age group, 35–74 years, US, 2003–2019**


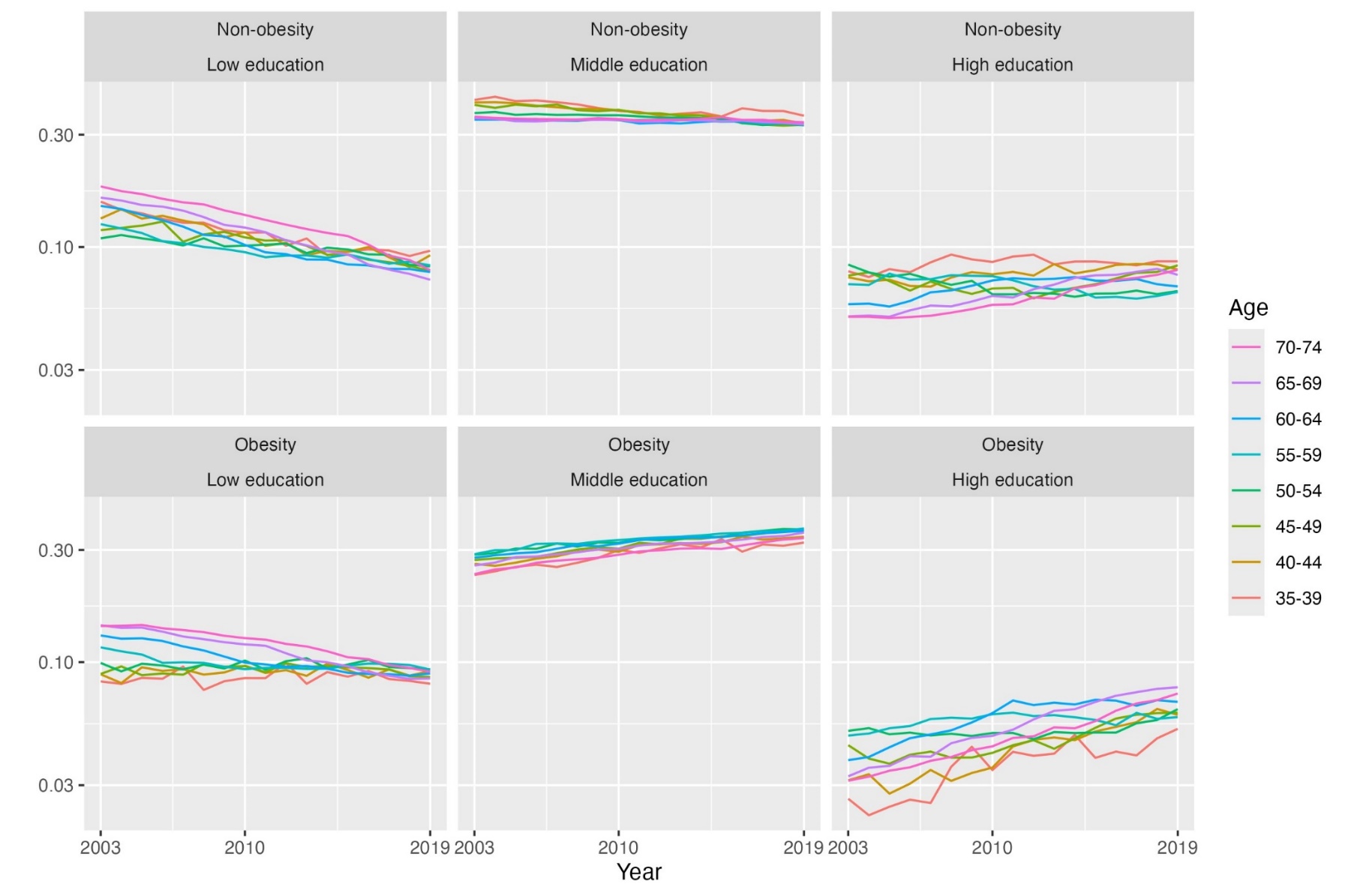


Source: NCHS data. Note: This chart shows *Pr(O,E|CVD)* in the conditional probability equation.

**Figure S4: Proportion of all CVD deaths that were within a specific obesity and education category, males, by age group, 35–74 years, US, 2003–2019**


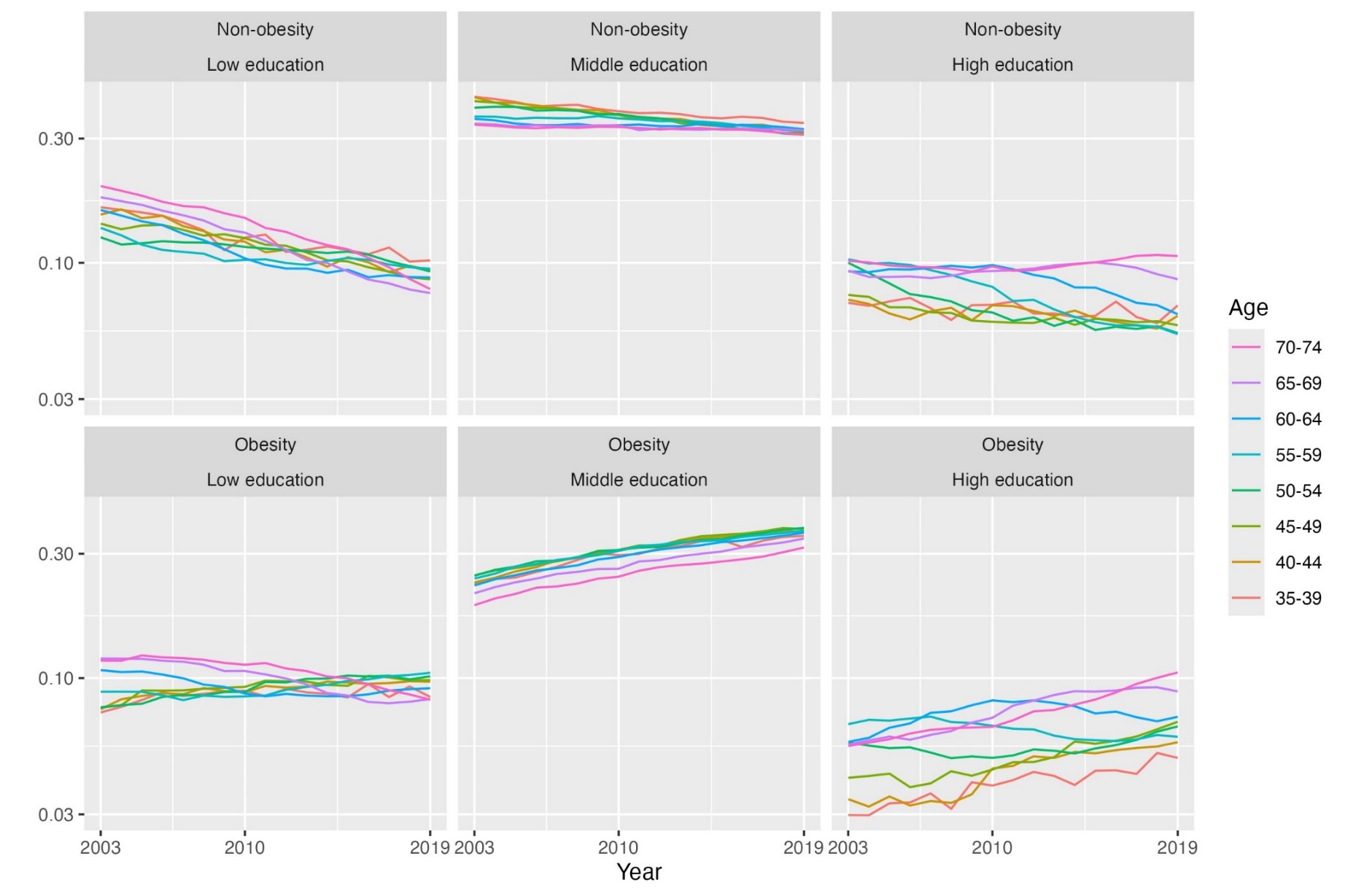


Source: NCHS data. Note: This chart shows *Pr(O,E|CVD)* in the conditional probability equation.
